# Survival benefit and biomarker of PD‐1 inhibitor combination therapy in first‐line of advanced biliary tract cancer: A retrospective study

**DOI:** 10.1002/cam4.6628

**Published:** 2023-11-06

**Authors:** Jingyi Guo, Qun Zhou, Mingzhen Zhou, Hengheng Dai, Lin Li, Yudong Qiu, Liang Mao, Baorui Liu, Jie Shen

**Affiliations:** ^1^ The Comprehensive Cancer Centre, Department of Oncology, Nanjing Drum Tower Hospital The Affiliated Hospital of Nanjing University Medical School Nanjing China; ^2^ Department of Oncology China Pharmaceutical University Nanjing Drum Tower Hospital Nanjing China; ^3^ Department of Radiology, Nanjing Drum Tower Hospital The Affiliated Hospital of Nanjing University Medical School Nanjing China; ^4^ Department of Oncology Nanjing Drum Tower Hospital Clinical College of Nanjing University of Chinese Medicine Nanjing China; ^5^ Department of Pathology, Nanjing Drum Tower Hospital The Affiliated Hospital of Nanjing University Medical School Nanjing China; ^6^ Department of Hepatopancreatobiliary Surgery, Nanjing Drum Tower Hospital The Affiliated Hospital of Nanjing University Medical School Nanjing China

**Keywords:** biliary tract cancer, biomarkers, combination therapy, PD‐1 inhibitors, survival

## Abstract

**Background:**

Immune checkpoint inhibitor (ICI) combination therapies have shown promise in the first‐line treatment of advanced biliary tract cancer (BTC). However, the best partner remains to be validated. Moreover, progress on biomarkers predicting the efficacy of ICI in BTC is slow. This study aimed to assess the efficacy and investigate reliable predictive biomarkers of programmed cell death protein‐1 (PD‐1) antibody combination therapy in the first‐line treatment of advanced BTC.

**Methods:**

Clinical data from patients with advanced BTC who received chemotherapy or anti‐PD‐1 combination therapy as first‐line were collected. The primary outcome was overall survival (OS). Biomarkers, including peripheral blood inflammation scores, genetic alterations, and tumor microenvironment were investigated.

**Findings:**

Sixty‐four patients were recruited and divided into four treatment groups: chemotherapy, anti‐PD‐1 plus chemotherapy, anti‐PD‐1 plus targeted therapy, and triple group (anti‐PD‐1 plus chemotherapy and targeted therapy). The median OS was 7.9, 11.3, 12.8, and 28.7 months, respectively. Compared to chemotherapy, mOS significantly prolonged in the triple group (*p* = 0.031). It showed that patients with five different peripheral blood inflammation scores had significantly prolonged mOS (*p* < 0.05). Genetic testing results suggested that patients with poor survival all had *TP53* mutations and higher levels of *KRAS* and *ERBB2* mutations. Low FOXP3/CD8 ratio was associated with prolonged OS (*p* = 0.029). With CD4‐low, CD8‐high, CD56‐positive, CD163‐high, FOXP3‐high and MPO‐high in TME as one factor, we calculated PLUS score according to the number of factors. The high‐PLUS (>2) group showed significantly superior OS (*p* = 0.003).

**Interpretation:**

First‐line anti‐PD‐1 combination therapy was superior to chemotherapy, and triple therapy significantly improved survival. Peripheral blood immune‐inflammation score, FOXP3/CD8 ratio, and PLUS have potential as biomarkers for predicting the efficacy of first‐line anti‐PD‐1 therapy in advanced BTC.

## 1 INTRODUCTION

Biliary tract cancers (BTC) are a group of low incidence hepatobiliary malignancies that include gallbladder carcinoma (GBC), intrahepatic cholangiocarcinoma (ICC), and extrahepatic cholangiocarcinoma (ECC). Incidence varies by region, with the highest incidence in Southeast Asia.^1^ Its morbidity and mortality are rising.^2^, ^3^ Surgery is the only treatment that may eradicate BTC, however, most patients are already advanced at the time of initial diagnosis, thus losing the opportunity for surgery.^4^ And there is a high rate of recurrence after surgery.^5^ Almost all patients end up on palliative medication.

Based on the ABC‐02 study,^6^ a chemotherapy regimen of gemcitabine combined with cisplatin (GP) is the standard first‐line treatment for advanced BTC, with a median overall survival (mOS) of approximately 1 year. Other new chemotherapy regimens have been tried, but the improvement in efficacy has not been remarkable and overall survival (OS) remains unsatisfactory.^7^ Hence, there is a need to explore new treatment methods.

ICIs are now widely used in clinical practice, with the most widely used being anti‐programmed cell death 1/ligand 1 (PD‐1/PD‐L1) antibodies. Their regimens in combination with chemotherapy or targeted therapy have been approved for first‐line treatment of various tumors, such as head and neck tumors, lung cancer and renal carcinoma. Anti‐PD‐1/PD‐L1 combination therapy has also been explored in the first‐line setting of BTC. Durvalumab (an anti‐PD‐L1 antibody) plus GP showed significantly prolonged OS compared to GP alone (12.8 vs. 11.5 months; HR: 0.80, 95% CI: 0.66–0.97, *p* = 0.021) in TOPAZ‐1 trial,^8^ and were recommended as one of the first‐line options for unresectable and metastatic BTC in the National Comprehensive Cancer Network guidelines 2022. The results of KEYNOTE‐966 were recently published, demonstrating that Pembrolizumab (an anti‐PD‐1 antibody) in combination with a GP regimen was significantly superior to GP alone in the first‐line treatment of advanced BTC (mOS: 12.7 vs. 10.9 months; HR: 0.83, 95% CI:0.72–0.95, *p* = 0.020).^9^ Several phase II/III clinical trials are also underway to evaluate the use of anti‐PD‐1 combination therapy in the first‐line setting for BTC, and there are few head‐to‐head comparative studies of various anti‐PD‐1 combination regimens versus standard chemotherapy alone in advanced BTC. Therefore, the application of specific treatment regimens in BTC remains to be elucidated.

Currently, there is a lack of favorable biomarkers for immunotherapy of BTC. Different studies have attempted to investigate possible biomarkers established in other tumor types, including PD‐L1 expression, tumor mutational burden (TMB), and microsatellite status.^10^ However, there is no definitive evidence that the aforementioned biomarkers help predict which patients are likely to benefit most from the use of ICIs.^11^ Although efforts have been made to reveal the genetic profile and tumor microenvironment (TME) of BTC to determine the prognostic value of ICIs, progress has been slow.

Therefore, we conducted this retrospective study to collect clinical data from patients with advanced BTC receiving chemotherapy or anti‐PD‐1 combination therapy as first‐line treatment for analysis to compare the survival benefit of different anti‐PD‐1 combination therapy versus chemotherapy. We also investigate the potential predictive biomarkers of survival for anti‐PD‐1 therapy.

## 2 METHODS

### 2.1 Study design

This was a single‐center, retrospective study conducted at Nanjing Drum Tower Hospital on the efficacy of different first‐line treatment regimens for patients with advanced BTC. We assessed four groups of patients: chemotherapy group, anti‐PD‐1 plus chemotherapy group, anti‐PD‐1 plus targeted therapy group, and triple group (anti‐PD‐1 plus chemotherapy and targeted therapy). Chemotherapy regimens mainly included gemcitabine plus cisplatin, oxaliplatin, or S‐1. There is no restriction on the type of anti‐PD‐1 antibody. Targeted drugs mainly include anlotinib and lenvatinib. The dosage of medication was chosen according to guideline recommendations as well as the patients' condition. This study used retrospective, anonymized clinical data, which were obtained after each patient had consented to treatment.

Advanced BTC was defined as unresectable BTC (pathologically proved by biopsy or surgical specimen, distant metastasis, multiple lesions) or relapses after radical surgery. Other eligibility criteria included an age of 18 years or older, an Eastern Co‐operative Oncology Group performance status (ECOG PS) score of 0–2 and no severe comorbidities. Patients whose treatment does no fall into any of these groups were excluded. The main baseline demographics and clinical characteristics included age, sex, ECOG PS score, pathology typing, metastases, prior treatment, and drug types.

Optional baseline biopsy specimens and blood samples were obtained from patients receiving anti‐PD‐1 antibodies for exploratory biomarker assessment. Exploratory endpoints included the association between survival and inflammation score, genetic variations, and immune microenvironment (TME).

### 2.2 Data collection and response assessment

Peripheral blood test results and clinical information were collected from the medical records of all enrolled patients within 1 week prior to treatment. Tumor evaluation was conducted based on computed tomography (CT) or magnetic resonance imaging (MRI). Radiologists reviewed imaging data and evaluated tumor response according to the Response Evaluation Criteria in Solid Tumors (RECIST) version 1.1 for solid tumor. Data were last updated on September 6, 2022. The primary outcome was OS. Secondary outcomes included progression‐free survival (PFS), objective response rate (ORR), and disease control rate (DCR). OS was the time from the initial treatment to death or cutoff date (which comes first). PFS was the duration from the beginning of treatment to disease progression. ORR was defined as the percentage of patients with a confirmed complete or partial response (CR/PR). DCR was the proportion of patients who have had a confirmed complete or partial response or stable disease (SD).

### 2.3 Inflammation score calculation

A total of eight inflammation scores were selected for analysis, including platelet‐to‐lymphocyte ratio (PLR), neutrophil‐to‐lymphocyte ratio (NLR), lymphocyte‐to‐C‐reactive protein ratio (LCR), lymphocyte‐to‐monocyte ratio (LMR), C‐reactive protein‐to‐albumin ratio (CAR), systemic immune‐inflammation index (SII), prognostic nutritional index (PNI), and pan‐immune‐inflammation value (PIV). The specific calculation of each inflammation score is shown in Table S1, and the optimal cutoff point for each score for survival prediction was determined using the X‐tile software (version 3.6.1).

### 2.4 Genetic analysis

Tumor tissues were collected pretreatment and at surgery for gene expression analysis. Gene expression was determined using next‐generation sequencing (NGS) at OrigiMed (Shanghai, China). Sequencing was based on the 706‐gene panel, which covers all coding exons of 706 genes associated with cancer and 64 selected introns of 77 genes frequently rearranged in solid tumors. Genes were captured and sequenced using the Illumina NextSeq 500 platform (Illumina Incorporated, San Diego, CA, USA), and peripheral blood from patients was used as a normal control sample for genomic analysis.

### 2.5 Immunohistochemical staining

For patients with available archival tissues, CD4, CD8, CD56, CD163, FOXP3, and MPO expression were assessed. Immunohistochemical staining was done by specialist pathologists and assessed quantitatively using the positive stained cell count method, whereby 10 <1/2 fields of view around the tumor nest were selected for counting positive stained cells under a 40× light microscope and averaged. According to previous study, immune cells were identified as either T‐helper cells (CD4^+^),^12^ regulatory T lymphocytes (FOXP3+),^12^ cytotoxic T lymphocytes (CD8^+^),^12^ natural killer (NK) cells (CD56^+^),^13^ M2 macrophages (CD163^+^)^14^ and granulocytes (MPO+).^15^ For statistical analyses, cases were assessed in groups using the optimal cutoff value of the X‐tile, defining those cases with positive stained cell counts above the cutoff value as the high infiltration group and those below the cutoff value as the low infiltration group. Overall, NK cell and B lymphocyte count numbers were very low; therefore, we grouped cases as having none (negative) or any number (positive) of infiltrating NK cells or B lymphocytes.

Following to Tanaka's study,^16^ we took CD4‐low, CD8‐high, CD56‐positive, CD163‐high, FOXP3‐high, and MPO‐high were taken as one factor and added up the number of factors for each case to become the PLUS score, which can thus be divided into 7 stages from 0 to 6. PLUS score was high for the score of 3–7 and low for the score of 0–2.

### 2.6 Statistical analysis

Continuous variables were presented as the mean ± standard deviation (mean ± SD) and between‐group differences were compared using ANOVA or Kruskal–Wallis test. Categorical variables were presented as a number with percentage. Chi‐squared or Fisher exact test was used to evaluate differences across the groups. PFS and OS analyses were conducted with Kaplan–Meier method and log‐rank test was used to identify variables that were statistically significantly associated with survival. Hazard ratios (HR) and 95% confidence intervals (CI) were measured. For all analyzes, *p* < 0.05 was considered to be statistically significant. SPSS V.25.0, GraphPad Prism V.9.2.0, and R version 4.3.1 were applied for data analysis and graph illustrating.

## 3 RESULTS

### 3.1 Patients

**FIGURE 1:**
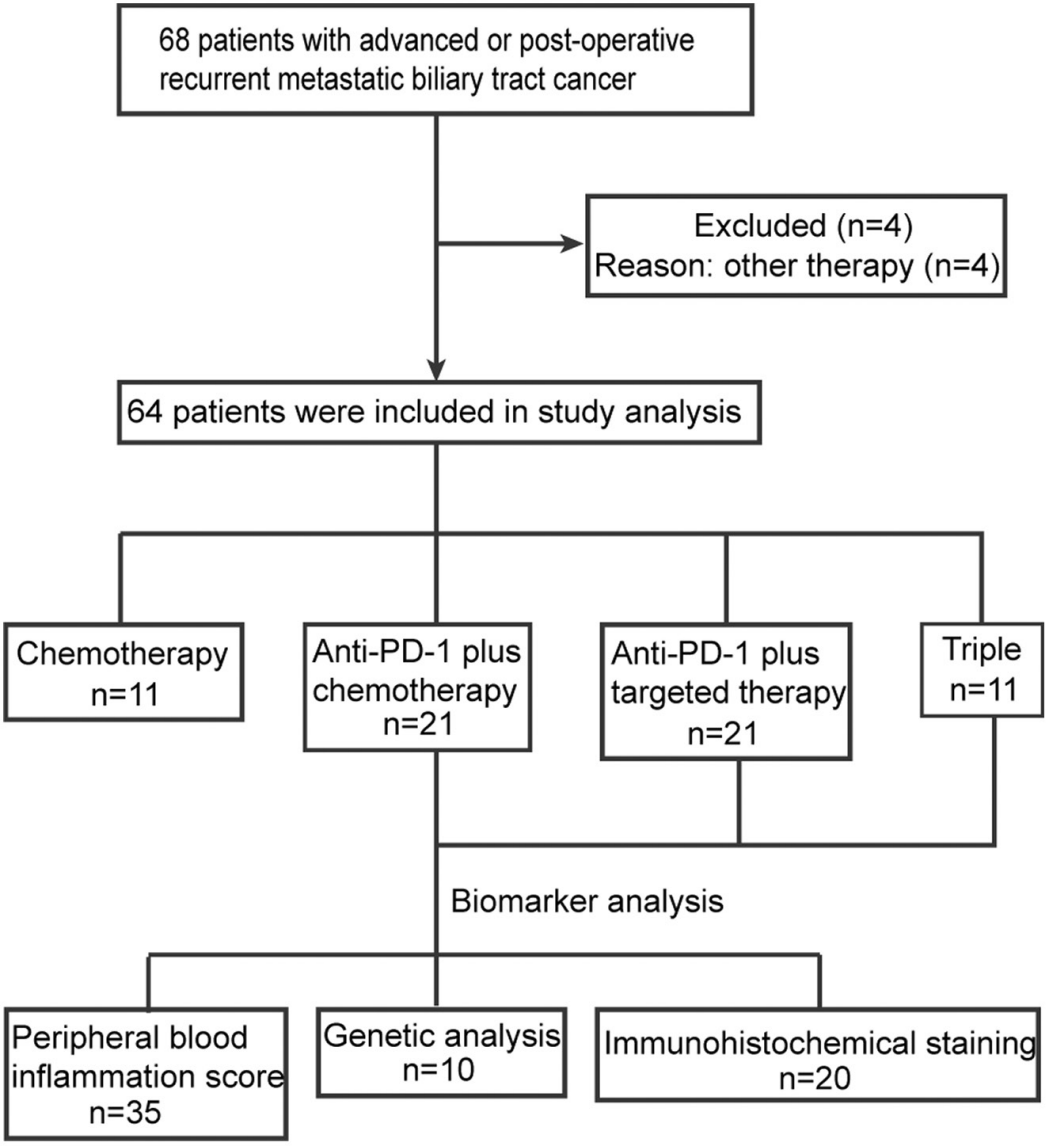
Flow chart of study design.

A total of 68 patients with histologically diagnosed BTC were screened from January, 2018, to July, 2022, and 64 patients who met the inclusion criteria were categorized into one of four treatment cohorts: 11 were in the chemotherapy group, 21 were in the anti‐PD‐1 plus chemotherapy group, 21 were in the anti‐PD‐1 plus targeted therapy group, and 11 were in the triple group (Figure 1). Baseline patient characteristics are shown in Table 1. The demographics and baseline characteristics were similar across the treatment groups, except for the BTC subtype, and targeted drugs. There is a largest proportion of gallbladder cancer patient in the chemotherapy group (54.5%). The anti‐PD‐1 plus targeted group has the highest percentage of patients with ICC (90.5%). ECC is most common in the triple group (18.2%). Lenvatinib had the highest frequency in both two groups of patients who used the targeted drug.

**TABLE 1:** Baseline characteristics.

Characteristics	C (*n* = 11)	A + C (*n* = 21)	A + T (*n* = 21)	T (*n* = 11)	*p*
Age					0.429
Mean ± SD	60.7 ± 7.2	56.5 ± 10.0	60.5 ± 10.9	61.1 ± 10.6	
Range	49–72	34–78	35–77	42–79	
Sex, %					0.636
Male	36.4%	42.9%	57.1%	54.5%	
Female	63.6%	57.1%	42.9%	45.5%	
ECOG, %					0.830
0	72.7%	81.0%	76.2%	90.9%	
1	27.3%	19.0%	19.0%	9.1%	
2	0	0	4.8%	0	
BTC subtype, %					0.003
GBC	54.5%	33.3%	0	36.4%	
ICC	45.5%	52.4%	90.5%	45.5%	
ECC	0	14.3%	9.5%	18.2%	
Distant metastasis, %					0.133
Yes	45.5%	76.2%	66.7%	90.9%	
No	54.5%	23.8%	33.3%	9.1%	
Previous surgery, %					0.818
Yes	36.4%	42.9%	52.4%	36.4%	
No	63.6%	57.1%	47.6%	63.6%	
Chemotherapy regimen, %					0.103
GS	36.4%	38.1%		0	
GP/GEMOX	27.3%	42.9%		63.6%	
Other	36.4%	19.0%		36.4%	
Anti‐PD‐1 antibody, %					0.335
Sintilima		28.6%	52.4%	36.4%	
Tislelizumab		14.3%	4.8%	0	
Toripalimab		33.3%	33.3%	18.2%	
Camrelizumab		14.3%	4.8%	36.4%	
Nivolumab		9.5%	4.8%	9.1%	
Targeted drug, %					
Anlotinib			28.6%	0	0.026
Lenvatinib			71.4%	81.8%	
Other			0	18.2%	

Abbreviations: A + C, anti‐PD‐1 plus chemotherapy group; A + T, anti‐PD‐1 plus targeted therapy group; C, Chemotherapy group; T, triple group.

### 3.2 Clinical outcomes

**FIGURE 2:**
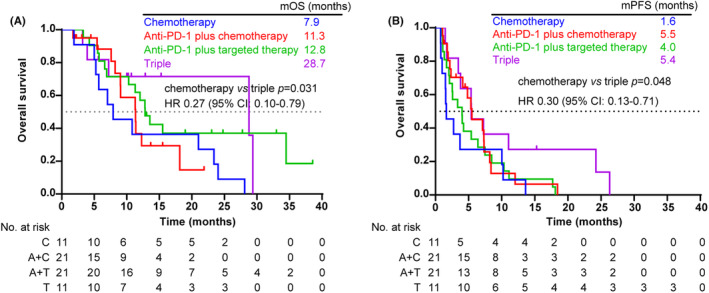
Survival benefit. (A) Kaplan–Meier estimate of overall survival. (B) Kaplan–Meier estimate of progression‐free survival. C: Chemotherapy group; A + C, anti‐PD‐1 plus chemotherapy group; A + T, anti‐PD‐1 plus targeted therapy group; T, triple group.

At the cutoff date (September 6, 2022), 59 (92.2%) PFS events and 41 (64.1%) deaths had occurred. The mOS was 7.9 months for the patient receiving chemotherapy, 11.3 months for the anti‐PD‐1 plus chemotherapy group, 12.8 months for those receiving anti‐PD‐1 plus targeted therapy, and 28.7 months for the triple group. OS was significantly improved with triple group compared with chemotherapy alone (HR 0.35, 95% CI: 0.13–0.95; *p* = 0.031, Figure 2A). Likewise, the median PFS was also significantly longer with patients receiving triple therapy (5.4 months) than those in the chemotherapy group (1.6 months) (HR 0.44, 95% CI: 0.18–1.10; *p* = 0.048, Figure 2B). The median PFS was 5.5 months in the anti‐PD‐1 plus chemotherapy group versus 4.0 months in the anti‐PD‐1 plus targeted therapy group.

**FIGURE 3:**
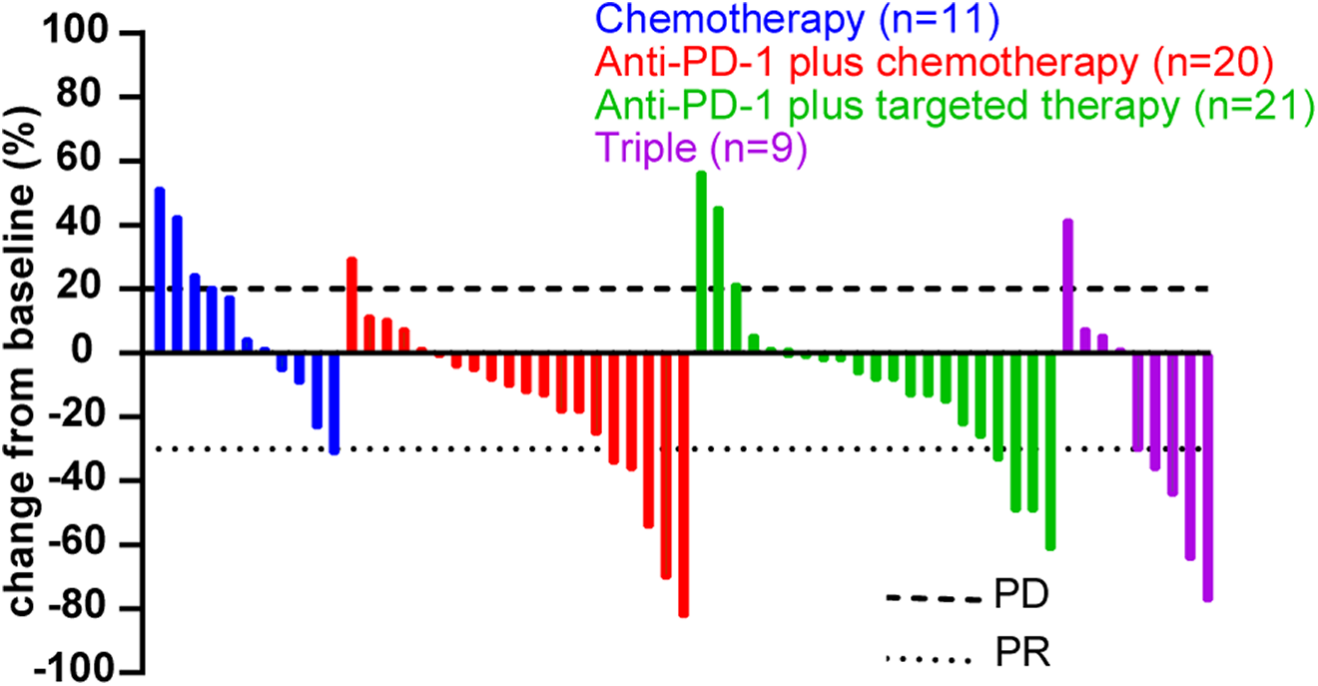
Waterfall plots of the percentage change from baseline.

Because three patients (1 in anti‐PD‐1 plus chemotherapy group, 2 in triple group) from out of town chose to have their imaging evaluation at a local hospital, only 61 patients were evaluated for tumor regression. The results are shown in Table 2. Among the four cohorts, the triple group showed the highest ORR (55.6%). However, the highest DCR (95%) was found in the anti‐PD‐1 plus chemotherapy group. Moreover, a two‐by‐two comparison revealed no significant difference between the groups. Figure 3 illustrates the changes in tumor size from baseline in 61 patients.

**TABLE 2:** Tumor response to treatment in each treatment group.

Response	C (*n* = 11)	A + C (*n* = 20)	A + T (*n* = 21)	T (*n* = 9)
CR	0	0	0	0
PR	1	5	4	5
ORR (%)	9.1 %	25.0%	19.0%	55.6%
SD	6	14	14	3
DCR (%)	63.6%	95.0%	85.7%	88.9%
PD	4	1	3	1

Abbreviations: A + C, anti‐PD‐1 plus chemotherapy group; A + T, anti‐PD‐1 plus targeted therapy group; C, Chemotherapy group; T, triple group.

### 3.3 Inflammation score

**FIGURE 4:**
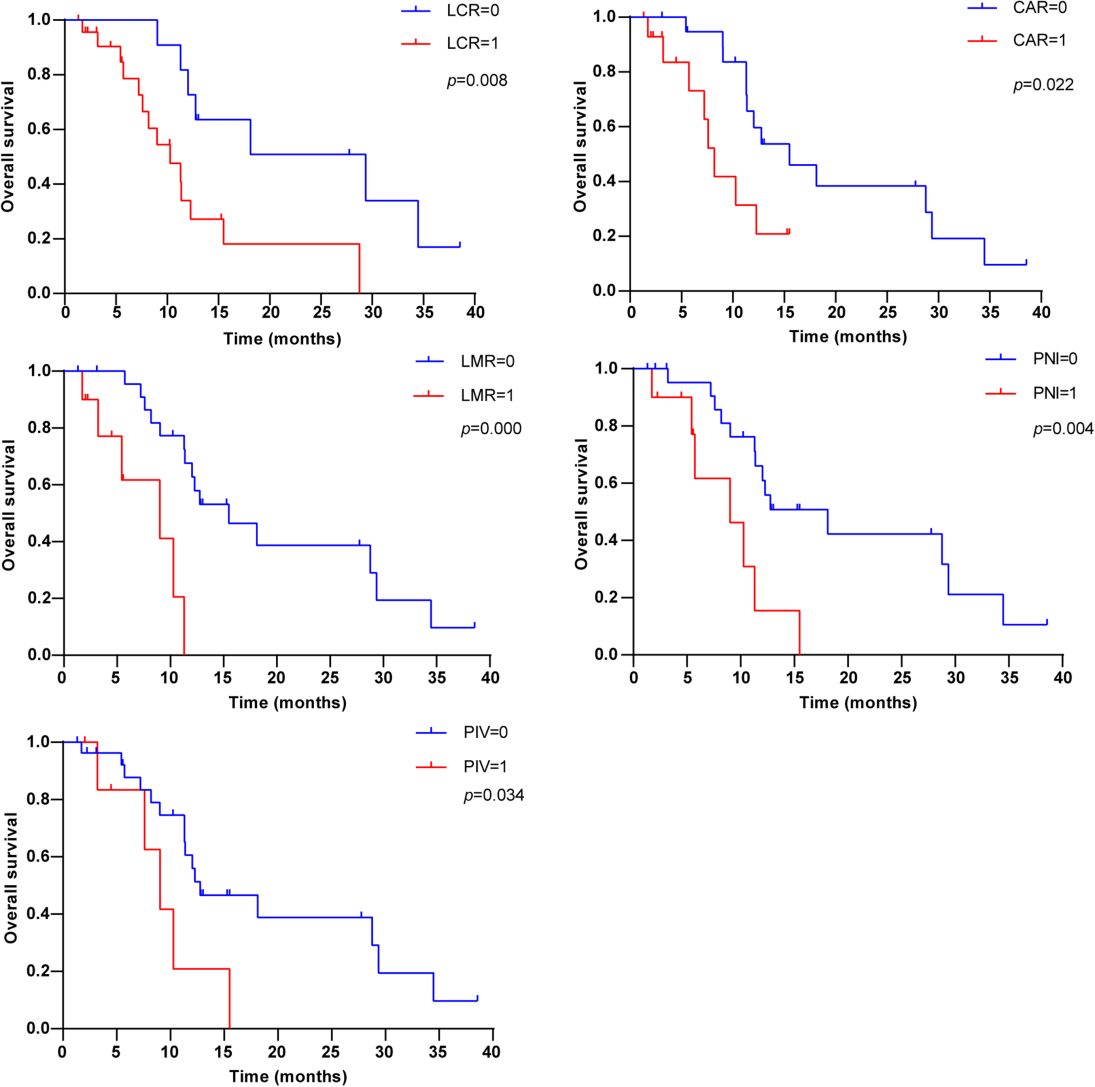
Kaplan–Meier estimates of overall survival in different peripheral blood inflammation score before treatment in patients receiving anti‐PD‐1.

To validate whether the inflammation score can be used as a biomarker to predict the efficacy of anti‐PD‐1 treatment, eight scores were selected for analysis. Of the 53 patients treated with anti‐PD‐1‐containing regimens in the first line, peripheral blood data were available for 35 patients. Demographic and clinical characteristics are summarized in Table S2. Using the optimal cutoff value derived from X‐tile, the LCR‐high (≥0.4), LMR‐high (≥2.5), CAR‐low (<0.18), PNI‐high (≥44.3), or PIV‐low (<480.9) patients demonstrated a significantly longer OS than the ones with different score (Figure 4). While no significant results could not be obtained for PLR, NLR, and SII (Figure S1).

**FIGURE 5:**
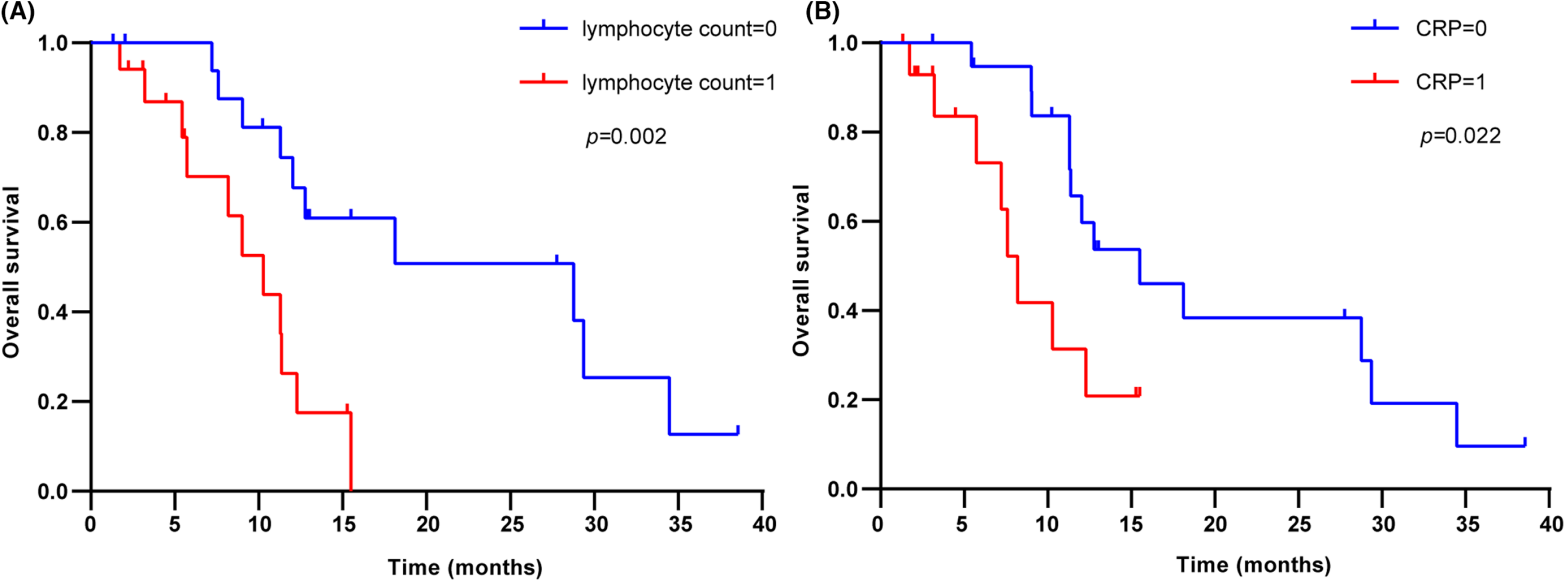
Kaplan–Meier estimate of overall survival with lymphocyte count score (A) and C‐reactive protein score (B).

We noted that multiple blood indicators were involved and duplicated in the five inflammation scores described above. Therefore, we proposed the hypothesis that these blood indicators could also be used to predict the efficacy of anti‐PD‐1. They were lymphocyte count, neutrophil count, monocyte count, platelet count, albumin, and CRP. Similarly, the X‐tile software was used to determine the optimal survival prediction cutoff values. The scores for peripheral blood indicators were calculated as shown in Table S3. The results showed that patients with high lymphocyte counts or low CRP had a longer OS (*p* = 0.002, *p* = 0.022, Figure 5). Results of other peripheral blood indicators were shown in Figure S2.

We then calculated the area under the curve (AUC) of the time‐dependent receiver operating characteristic (ROC) curves at 6, 12, 18, and 24 months of OS of the above obtained seven biomarkers significantly associated with OS to compare their prognostic value, and the values are provided in Table S4. The PNI score and Lymphocyte count consistently had higher values compared to other biomarkers beyond the 12‐month time point, suggesting that the PNI score, and Lymphocyte count may be better predictors of OS.

### 3.4 Genetic alterations

**FIGURE 6:**
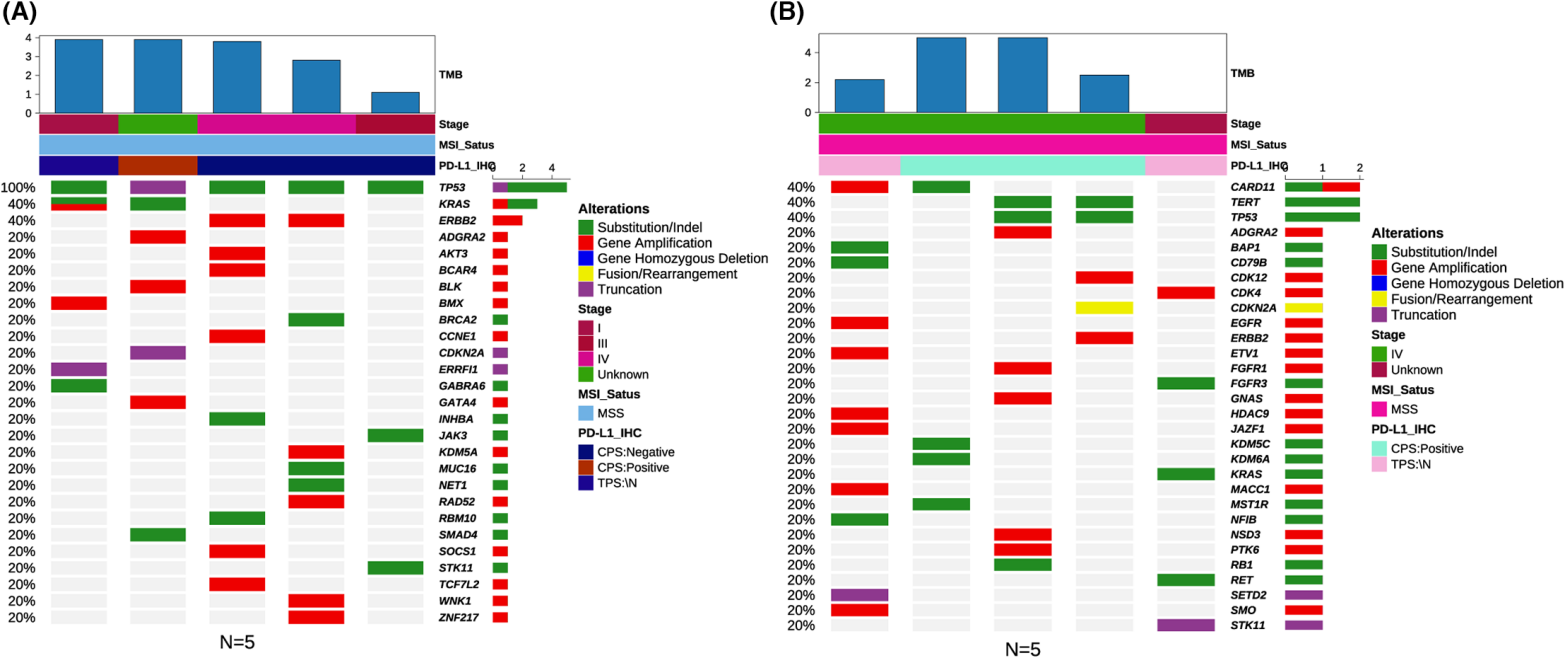
Distribution of genetic variations in poor survival (A) and good survival (B).

To investigate whether the genetic mutations carried by the patients had an impact on the efficacy of anti‐PD‐1 treatment, NGS was performed on tissue specimens from the patients to detect gene expression. Given the small sample size, we selected the five patients with the longest OS from the available patients as the “good survival” group and the five patients with the shortest OS as the “poor survival” group. And the OS data showed that there was indeed a significant difference in survival between the two group (Figure S3). As shown in the Figure 6, all five patients with poor survival carried *TP53* mutations, and the proportion of *KRAS* mutations (2/5) and *ERBB2* amplification (2/5) were higher than those of patients with good survival (1/5, 1/5). Unfortunately, these mutations were not significantly associated with OS. Other genetic changes in the good survival group were *CARD11* (2/5), *TRET* (2/5), and *TP53* (2/5).

### 3.5 Cell infiltration in TME

**FIGURE 7:**
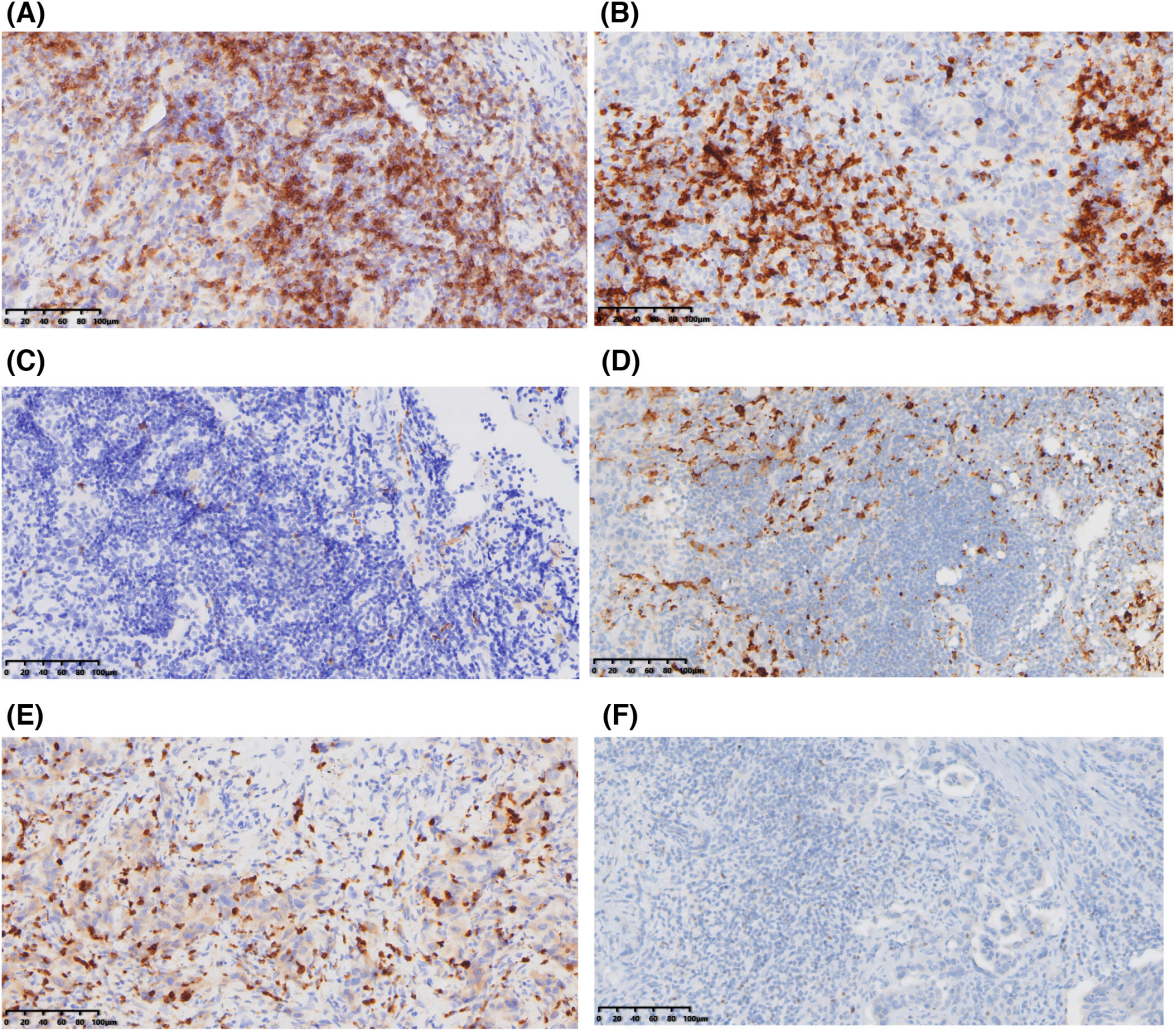
Representative immunohistochemical images of CD4 (A), CD8 (B), CD56 (C), CD163 (D), MPO (E), and FOXP3 (F). Brown means positive.

**FIGURE 8:**
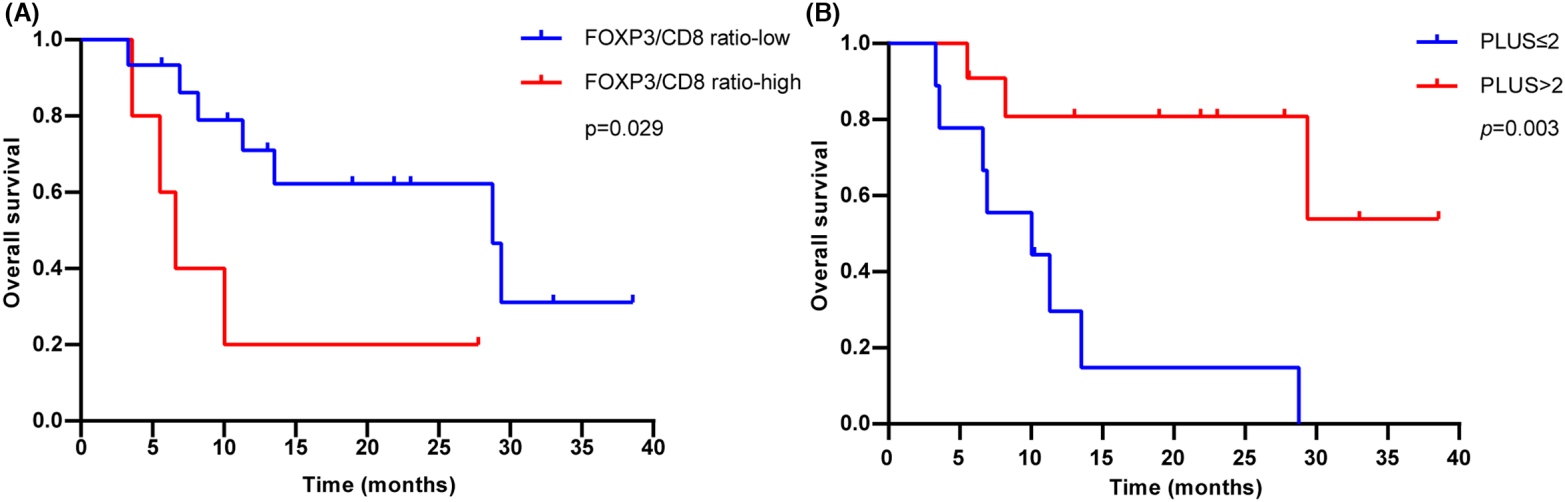
Kaplan–Meier estimates of overall survival with FOXP3/CD8 ratio (A) and PLUS score (B).

To understand the influence of immune cells infiltration into TME on the efficacy of anti‐PD‐1 combination therapy, tissue samples from 20 patients treated with anti‐PD‐1 were assessed. Representative images of these tissues stained for CD4, CD8, CD56, CD163, and MPO are shown in Figure 7. Expression of markers in TME was evaluated with OS. Results showed that there was no significant difference of any markers (Figure S4). Moreover, low FOXP3/CD8 ratio (≤0.27) was associated with improve OS (median: 28.7 vs. 6.6 months, HR 0.29, 95% CI: 0.05–1.57; *p* = 0.029) (Figure 8A).

In Tanaka's study, he integrated CD8‐high, CD4‐high, FOXP3‐high, and CD68‐low into a score called Immunoscore and divided BTC patients into two groups based on their scores, finding that patients in the high Immunoscore group had significantly superior OS (*p* = 0.001).^16^ We thus presumed that those factors with higher values of OS in the present study (CD4‐low, CD8‐high, CD56‐positive, CD163‐high, FOXP3‐high, and MPO‐high) could be integrated into a similar score named PLUS. The PLUS score was made up of the number of factors in each case. We then divided the patients into two groups according to whether the PLUS score was greater than 2 to analyze its relationship with OS, showing that patients with high PLUS (>2) had significantly superior OS than those with low PLUS (median: not reached vs. 10.0 months, HR 0.18, 95% CI: 0.05–0.64; *p* = 0.003) (Figure 8B).

## 4 DISCUSSION

BTC has long been regarded as a rare disease with poor prognosis and limited treatment options. To our knowledge, this is the first study to compare the efficacy of three anti‐PD‐1 combination therapies with chemotherapy in the first‐line setting of BTC in the real world. In addition, this study explored multiple biomarkers in patients with advanced BTC receiving antiPD‐1, including peripheral blood inflammation scores, genetic alterations, and immune cell infiltration in TME. The results showed that anti‐PD‐1 combination therapy has a potent anti‐tumor effect in advanced BTC, with better PFS and OS than chemotherapy, and the effect of the triple combination regimen is even more significant, with a mOS of 28.7 months, mPFS of 5.4 months. Multiple inflammation scores have the potential to predict the efficacy of anti‐PD‐1. More convenient blood results (lymphocyte count and CRP) have also been shown to correlate significantly with OS. FOXP3/CD8 ratio and PLUS composed of several immune cells were significantly associated with survival after anti‐PD‐1 therapy.

Two phase 2 clinical trials evaluating first‐line use of camrelizumab in combination with chemotherapy in patients with advanced BTC achieved mOS of 11.8^17^ and 12.4 months,^18^ respectively, and in our present retrospective analysis, a shorter mOS of 11.3 months was obtained. The difference between tumor subtypes and outcome has been reported. Li et al. suggested that GBC and ICC had better outcome compared to ECC.^19^ Compared to the two studies mentioned above, in our anti‐PD‐1 plus chemotherapy group that had a higher proportion of ECC. A previous Phase 2 trial reported a mOS of 17.7 months for patients with advanced BTC with lenvatinib combined with anti‐PD‐1 antibodies,^20^ but in this study, the mOS was only 12.8 months. Our study showed that 66.7% of the patients in this group had distant metastases, which means a worse prognosis. A study investigating the efficacy of toripalimab, lenvatinib in combination with oxaliplatin and gemcitabine as first‐line treatment were presented. Thirty patients with advanced ICC were enrolled. Remarkably, the median OS was 22.5 months.^21^ And we get a longer mOS in the real world at 28.7 months. These results suggest that anti‐PD‐1 in combination with chemotherapy and targeted therapy holds promise for first‐line treatment in advanced BTC. Overall, our results suggested that anti‐PD‐1 combination therapy had good promise as first‐line treatment for advanced BTC. There are also relevant large‐scale phase III trials currently underway, looking forward to their results.

There is growing evidence that certain cytotoxic chemotherapeutic agents have various immunostimulatory effects. Chemotherapy can upregulate the expression of major histocompatibility complex‐I (MHC‐I) and immune checkpoints in tumor cells, induce immunogenic cell death of tumor cells, enhance the function of immune cells as well as increase cytokine production, thereby reprogramming the tumor immune microenvironment, and enhancing anti‐tumor immunity in synergy with immunotherapy.^22^ Moreover, angiogenesis is critical in tumor survival and proliferation and resistance. Recently, it has been suggested that anti‐angiogenesis could modulate the tumor immune microenvironment. Angiogenesis factors suppress tumor immune responses by negatively affecting antigen‐presenting cells and effector T cells and enhancing the action of immunosuppressive cells such as regulatory T cells (Treg) and myeloid‐derived suppressor cells (MDSC).^23^ In theory, combination chemotherapy, immunotherapy, and anti‐angiogenic targeted therapy can work through different anti‐tumor pathways. All of the patients in the chemotherapy group received anti‐PD‐1 combination therapy as their second‐line treatment, but their survival remained shorter than that of patients who received the triple regimen in the first line. Notably, although not statistically different, the ORR in the triple group was more than six times higher than that of the chemotherapy group (55.6% vs. 9.1%). Similar to the findings of Zhu's study, early use of immunotherapy and chemotherapy combined with targeted therapy is recommended for patients who are able to tolerate it.^24^ Additionally, a recent meta‐analysis of the efficacy of anti‐PD‐1/PD‐L1 in advanced BTC showed that median OS was longer in the first‐line settings than in the second‐line settings or beyond.^25^

Peripheral blood inflammation scores have great potential in predicting the efficacy of immunotherapy and have been reported in a variety of tumors, such as hepatocellular carcinoma,^26^ non‐small cell lung cancer,^27^ and pancreatic cancer.^28^ Several studies have shown that inflammation scores can be used as a predictor of response to anti‐PD‐1 in patients with BTC.^29^, ^30^, ^31^ Given the small size of our sample, we chose inflammation scores that divided the patients into two groups from Yang’ study.^31^ In addition, we selected an inflammation score PIV involving four peripheral blood counts, which has never been validated in BTC. Of these eight inflammation scores we selected, LCR, LMR, CAR, PNI, and PIV were shown to significantly differentiate patients' OS (Figure 4). Moreover, we also analyzed the peripheral blood indicators involved in these scores and found that lymphocyte count and CRP were significant influences on OS. The patients with high lymphocyte count or low CRP had a longer OS. CRP has been reported as a predictor of prognosis in patients with intrahepatic cholangiocarcinoma treated with anti‐PD‐1 therapy.^31^ Our study expanded the results to BTC. Considering the small sample size of the current study, we intend to expand the sample size to further validate. Lymphocytes have a powerful anti‐tumor immune function and can inhibit the progression of many tumors. The lymphocyte count reflects the general state of the body's immune function. Elevated lymphocyte levels have been reported to be associated with a good prognosis of malignant tumors.^32^ To compare the performance of these peripheral biomarkers, we calculated their AUROC at 6, 12, 18, 24 months of OS. The results showed that PNI and lymphocyte count were better predictors of survival after first‐line anti‐PD‐1 treatment in patients with advanced BTC. The PNI score is calculated from albumin and lymphocyte counts and is a simple method for the assessment of the immune‐nutritional status of cancer patients. The efficacy of immunotherapy and long‐term outcomes in malignancies have been linked to nutrition and immune status. A recent meta‐analysis showed that PNI might be an effective biomarker of tumor response and prognosis of advanced cancer patients with ICIs,^33^ which is consistent with our findings. Our study suggests that not only peripheral blood inflammation scores, but also certain more convenient peripheral blood indicators have the potential to predict the prognosis of patients with advanced BTC receiving anti‐PD‐1 therapy. We will validate these results in a larger study.

The TME plays an important role in the development of tumor and can influence the clinical outcome of immunotherapy.^34^ Its role as a predictor of the response to ICIs is under evaluation in BTC. Most previous studies have focused on T lymphocytes and/or macrophages.^16^, ^35^, ^36^ We therefore selected representative indicators of multiple cells (including T cells, NK cells, M2‐macrophages, and granulocytes) for immunohistochemical staining to explore the prognostic role of TME in patients with BTC receiving anti‐PD‐1 therapy. Our results suggested that CD4‐low, CD8‐high, CD56‐positive, CD163‐high, FOXP3‐high, and MPO‐high had longer OS numerically (Figure S4). The results for individual indicators are not satisfactory. Considering that there are interactions between immune cells in TME, we tried to combine several indicators and analyze them together. Surprisingly, we found that patients with a low FOXP3/CD8 ratio have significantly longer OS (Figure 8A). In addition, inspired by Tanaka's research,^16^ we combined factors for longer OS, including CD4‐low, CD8‐high, CD56‐positive, CD163‐high, FOXP3‐high, and MPO‐high into an PLUS based on the effect of each marker on OS (Figure S4). The number of factors included in each case is the specific score. We then divided the patients into two groups using a cutoff of 2 scores. The results showed that patients with high PLUS had significantly longer OS compared to those with low PLUS (Figure 8B). This result requires a larger sample size for further validation. In any case, we consider it necessary to carry out a comprehensive analysis of the immune cells in TME.

This study has several limitations. First, selection bias and recall bias cannot be excluded due to its retrospective nature. Second, the small sample size may be insufficient to perform a statistically analysis. Third, there is no mandatory restriction for the varieties of drugs. The different efficacies of chemotherapeutic agents, anti‐PD‐1 antibodies, and targeted agents have been reported, and this factor may make it difficult to analyze the accurate efficacy of specific treatment protocols. Although these shortcomings somewhat undermine the validity and reliability of this study, real‐world data still can be used as a reference for the selection of clinical treatment strategies.

## 5 CONCLUSION

In summary, our results suggest that first‐line anti‐PD‐1 plus chemotherapy and targeted therapy in patients with metastatic or recurrent BTC significantly improve OS and PFS compared with chemotherapy alone. Pretreatment levels of peripheral hematologic factors, including increased LCR, LMR and PNI and decreased CAR and PIV were associated with longer OS and may be developed as potential predictors. We found that high PLUS and low ratio of FOXP3/CD8 had significantly longer OS, indicating that the evaluation of infiltrating immune cells in TME was useful to predict the efficacy of anti‐PD‐1 in advanced BTC.

## AUTHOR CONTRIBUTIONS

**Jingyi Guo:** Conceptualization (equal); data curation (equal); formal analysis (equal); writing – original draft (equal); writing – review and editing (equal). **Qun Zhou:** Data curation (equal); formal analysis (equal). **Mingzhen Zhou:** Data curation (equal); formal analysis (equal). **Hengheng Dai:** Formal analysis (equal). **Lin Li:** Data curation (equal); formal analysis (equal). **YuDong Qiu:** Formal analysis (equal); methodology (equal); writing – review and editing (equal). **Liang Mao:** Writing – review and editing (equal). **Baorui Liu:** Conceptualization (equal); methodology (equal). **Jie Shen:** Conceptualization (equal); methodology (equal); writing – review and editing (equal).

## FUNDING INFORMATION

This research did not receive any specific grant from funding agencies in the public, commercial, or not‐for‐profit sectors.

## CONFLICT OF INTEREST STATEMENT

All authors declare no conflict of interest.

## ETHICS STATEMENT

This retrospective study was approved by the Medical Ethics Committee of Drum Tower Hospital affiliated with Nanjing University Medical School (2023‐215‐01), and the requirement to obtain informed written consent was waived. The study was performed in accordance with the Declaration of Helsinki.

## Supporting information


Figure S1.

Figure S2.

Figure S3.

Figure S4.

Table S1.

Table S2.

Table S3.

Table S4.

## Data Availability

All data relevant to the study are in the text or uploaded to the supplementary material.
