# Supplementary figures and images for: Survival benefit and biomarker of PD‐1 inhibitor combination therapy in first‐line of advanced biliary tract cancer: A retrospective study
Source: Cancer Med. 2023 Nov 6;12(22):20699–711. doi: 10.1002/cam4.6628 (PMC10709733; doi:10.1002/cam4.6628)

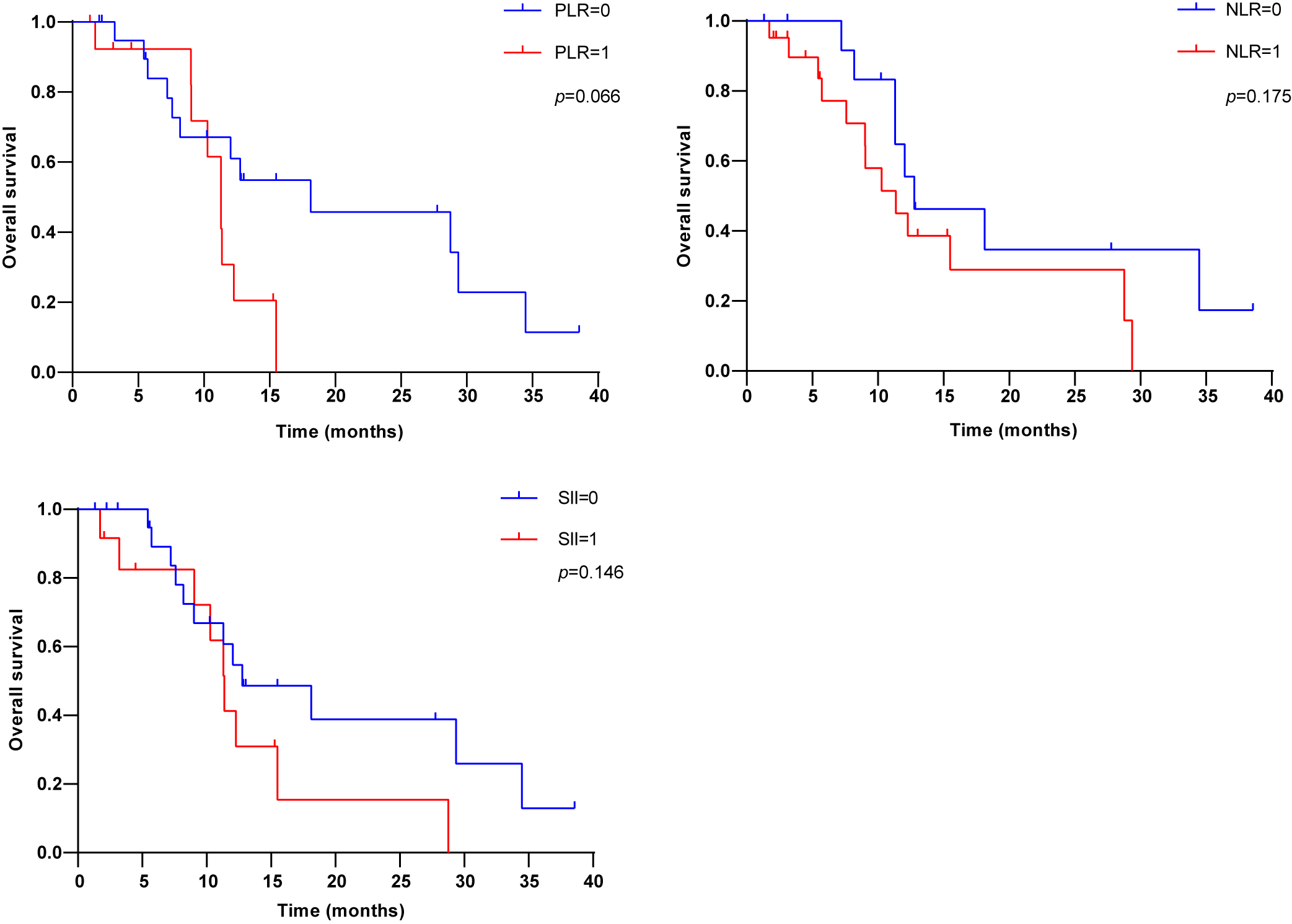

Supplement: Supplementary file 1 — Figure S1. [file CAM4-12-20699-s007.tif]

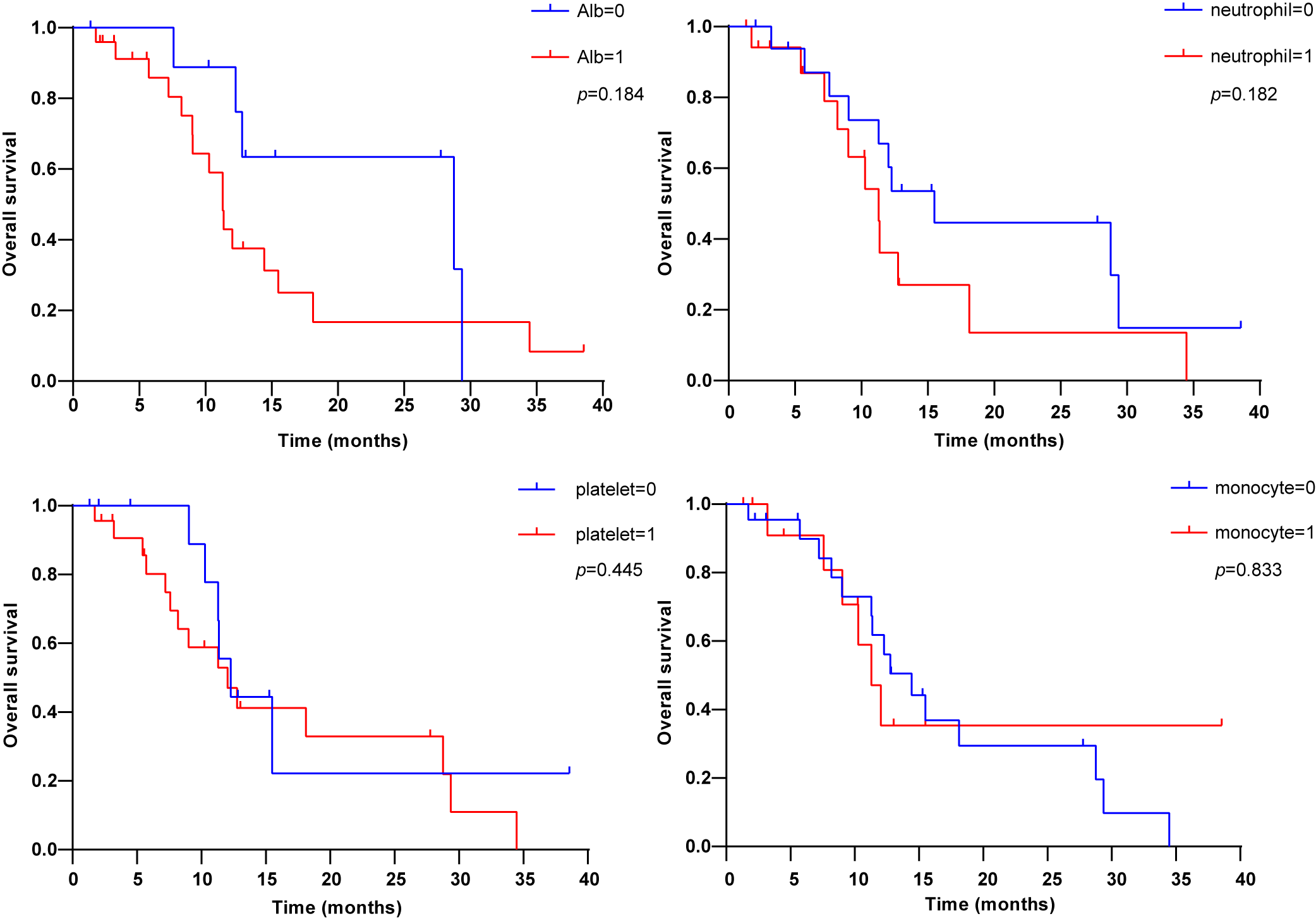

Supplement: Supplementary file 2 — Figure S2. [file CAM4-12-20699-s004.tif]

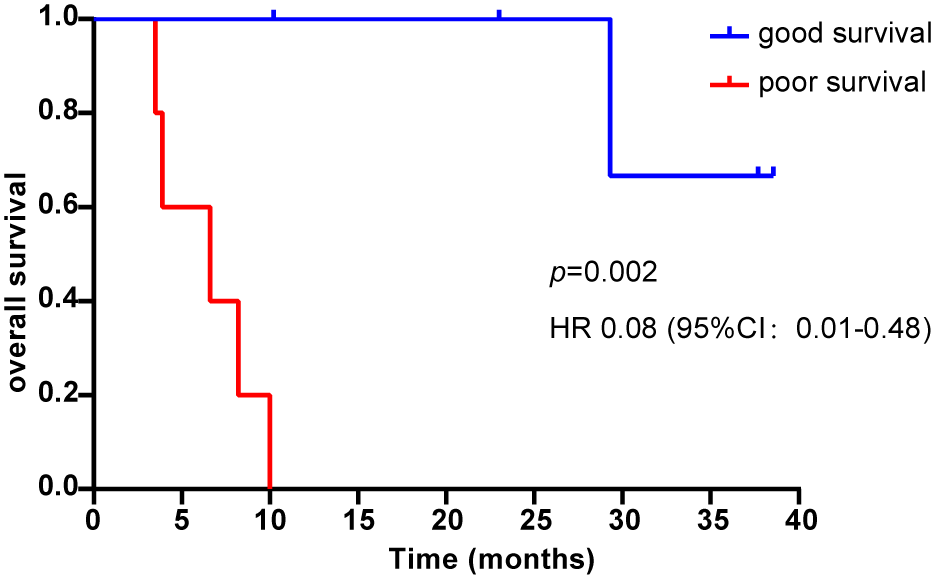

Supplement: Supplementary file 3 — Figure S3. [file CAM4-12-20699-s002.tif]

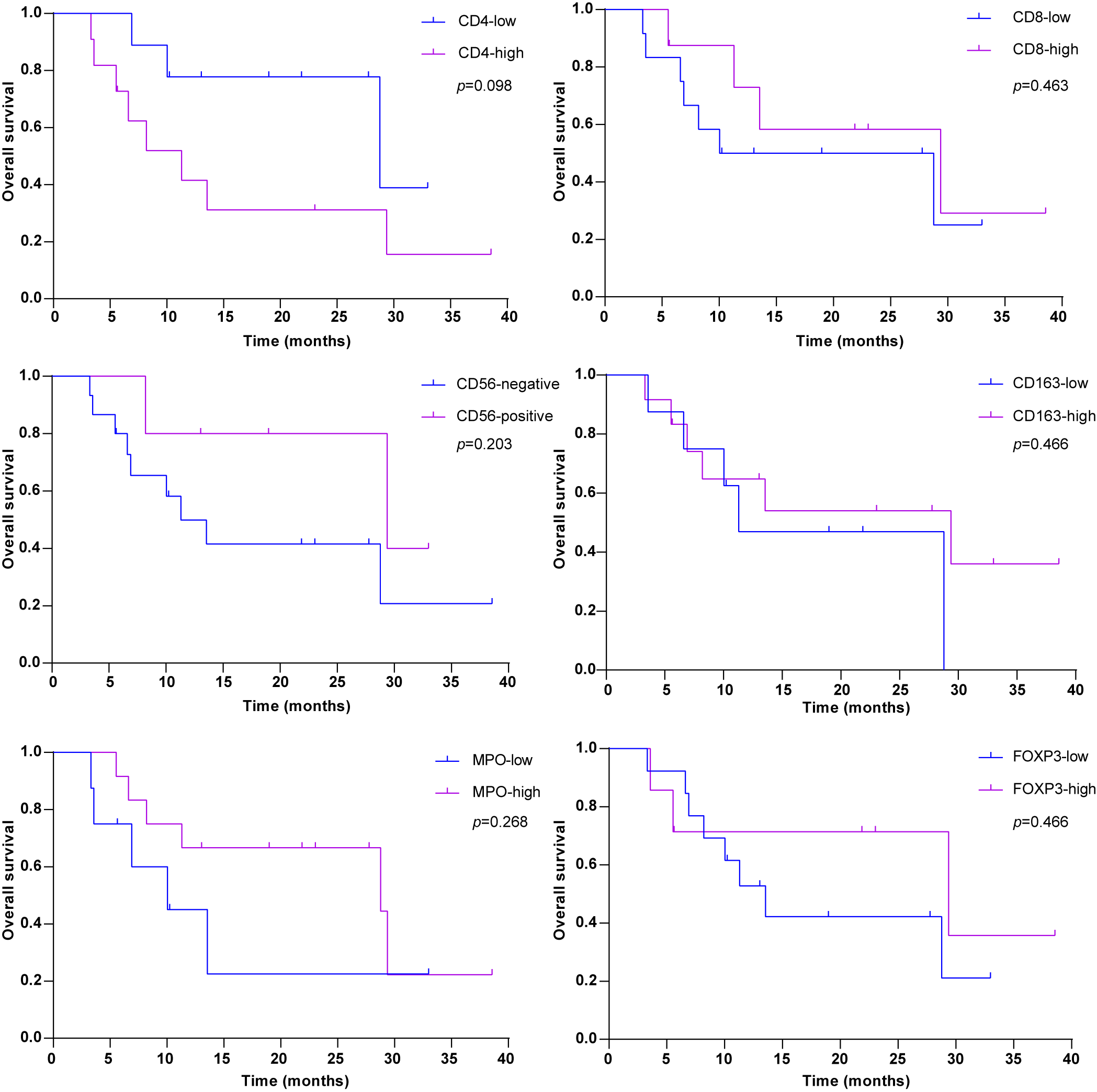

Supplement: Supplementary file 4 — Figure S4. [file CAM4-12-20699-s005.tif]

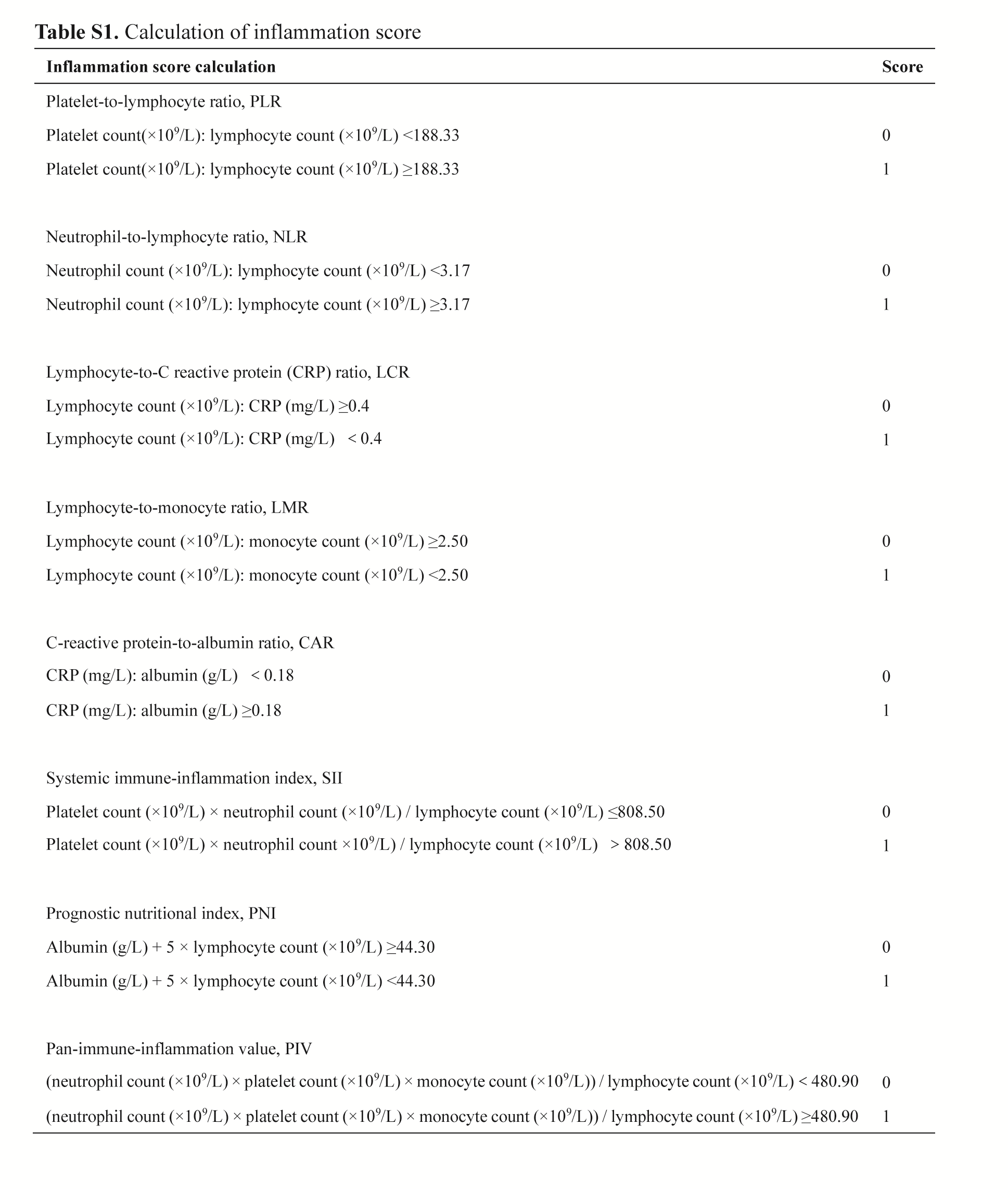

Supplement: Supplementary file 5 — Table S1. [file CAM4-12-20699-s001.tif]

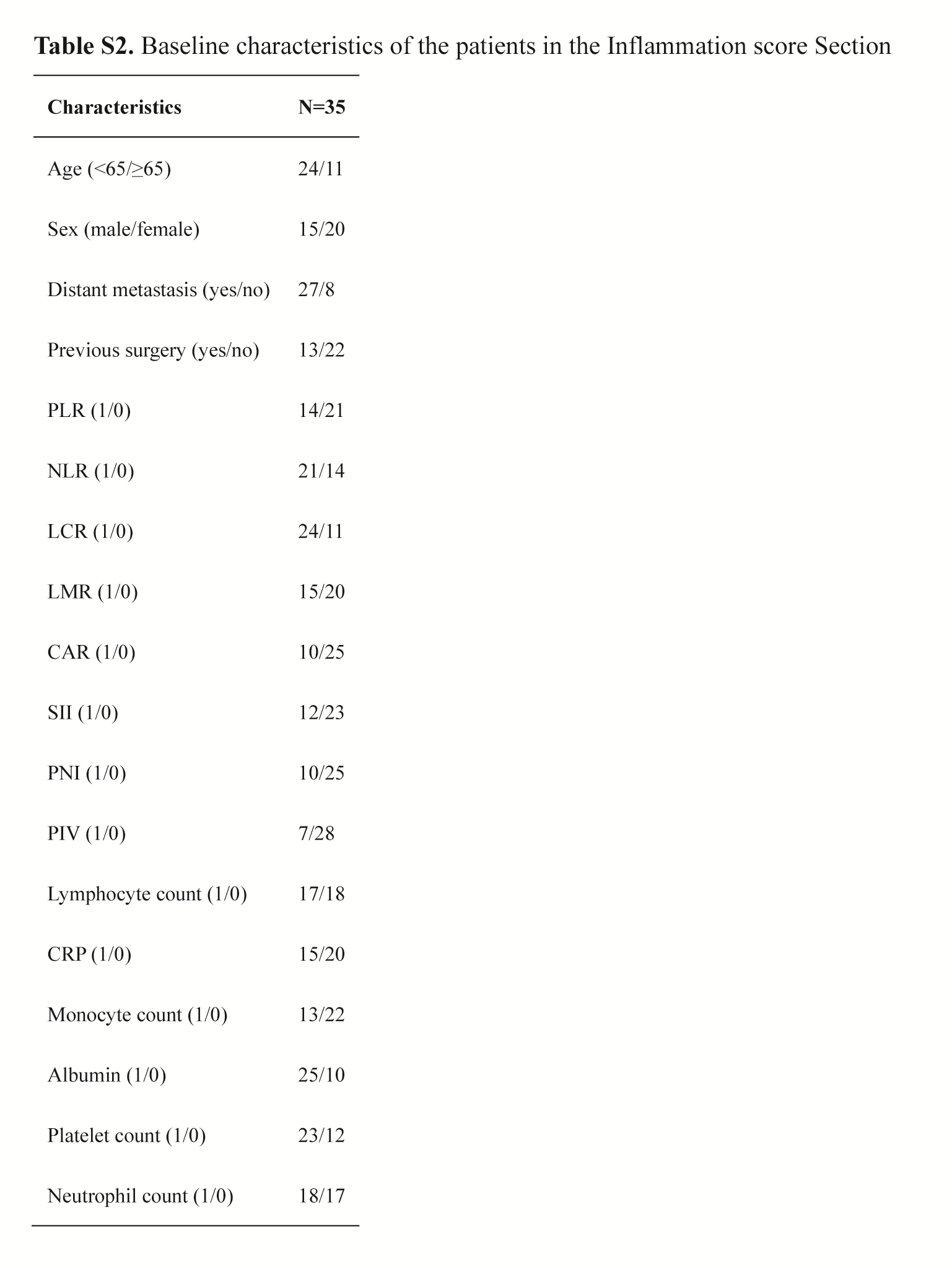

Supplement: Supplementary file 6 — Table S2. [file CAM4-12-20699-s003.tif]

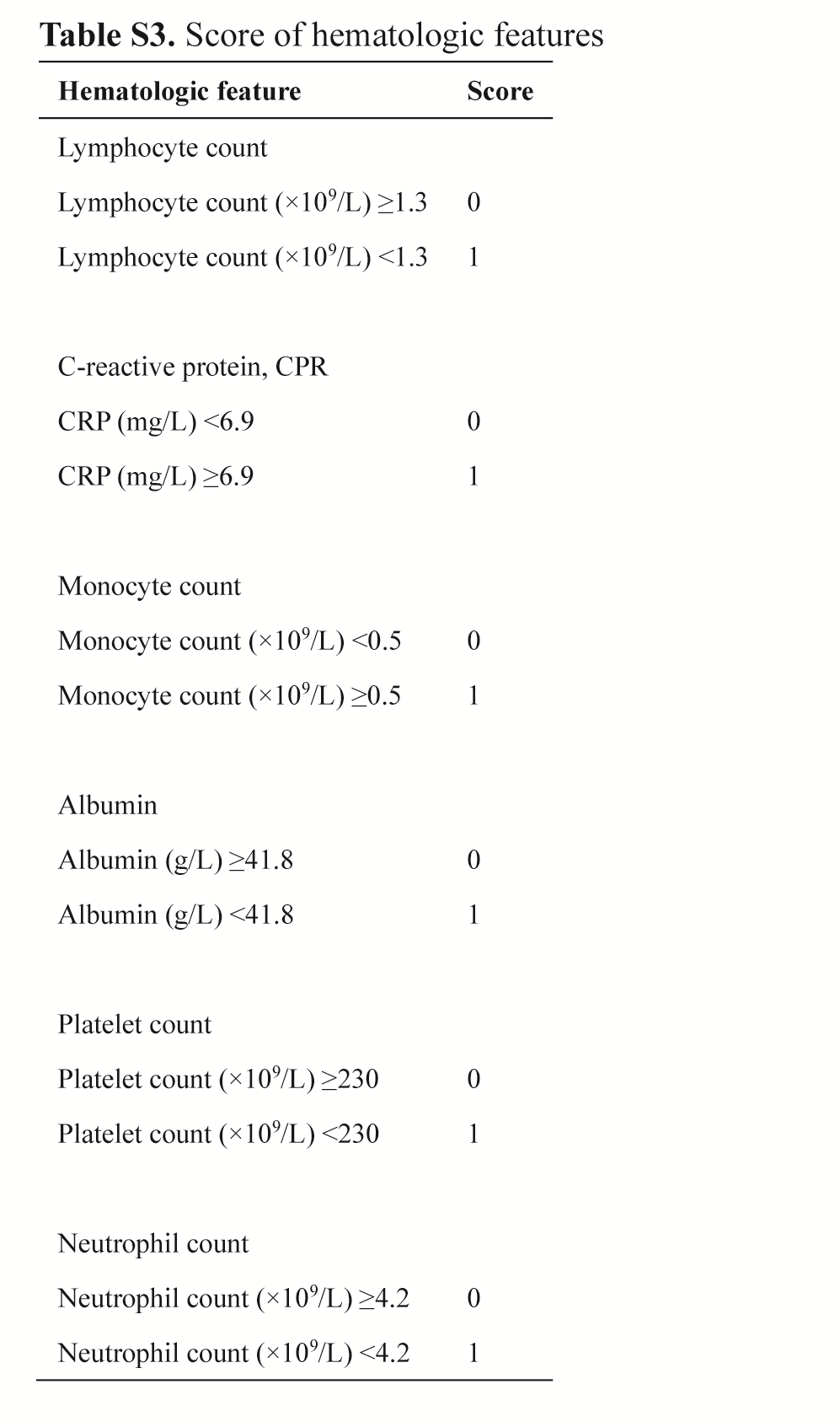

Supplement: Supplementary file 7 — Table S3. [file CAM4-12-20699-s006.tif]

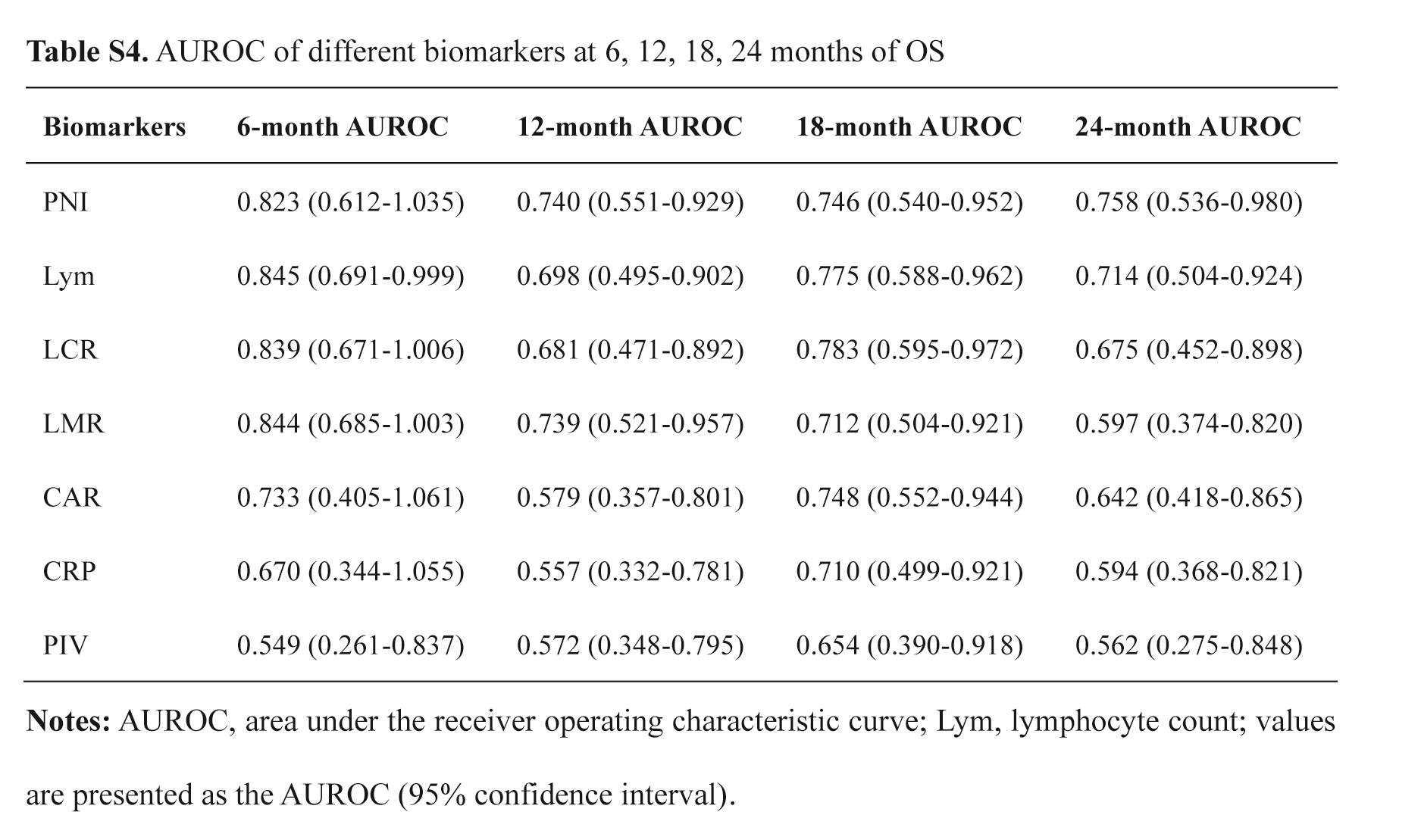

Supplement: Supplementary file 8 — Table S4. [file CAM4-12-20699-s008.tif]
